# Endophilin A2 promotes HER2 internalization and sensitivity to trastuzumab-based therapy in HER2-positive breast cancers

**DOI:** 10.1186/s13058-017-0900-z

**Published:** 2017-10-03

**Authors:** Tomas Baldassarre, Peter Truesdell, Andrew W. Craig

**Affiliations:** 1 Department of Biomedical and Molecular Sciences, Queen’s University, Kingston, Ontario Canada; 2 Cancer Biology & Genetics Division, Queen’s Cancer Research Institute, Kingston, Ontario Canada

**Keywords:** HER2 breast cancer, HER2 internalization, Trastuzumab, T-DM1, Endophilin

## Abstract

**Background:**

Human epidermal growth factor receptor-2 (HER2) is amplified and a clinical target in a subset of human breast cancers with high rates of metastasis. Targeted therapies involving the antibody trastuzumab and trastuzumab-emtansine (T-DM1) have greatly improved outcomes for HER2-positive (HER2+) breast cancer patients. However, resistance to these targeted therapies can develop and limit their efficacy. Here, we test the involvement of the endocytic adaptor protein endophilin A2 (Endo II) in HER2+ breast cancer models, and their responses to treatments with trastuzumab and T-DM1.

**Methods:**

Endo II expression in human breast tumors and lymph node metastases were analyzed by immunohistochemistry. Stable silencing of Endo II was achieved in HER2+ cancer cell lines (SK-BR-3 and HCC1954) to test Endo II effects on HER2 levels, localization and signaling, cell motility and tumor metastasis. The effects of Endo II silencing on the responses of HER2+ cancer cells to trastuzumab or T-DM1 treatments were tested using real-time cell motility and cytotoxicity assays.

**Results:**

High Endo II protein expression was detected in HER2-positive tumors, and was linked to worse overall survival in node-positive HER2+ breast cancers at the mRNA level. Stable silencing of Endo II in HER2+ cell lines led to elevated levels of HER2 on the cell surface, impaired epidermal growth factor-induced HER2 internalization, and reduced signaling to downstream effector kinases Akt and Erk. Endo II silencing also led to decreased migration and invasion of HER2+ cancer cells in vitro, and impaired lung seeding following tail vein injection in mice. In addition, Endo II silencing also impaired HER2 internalization in response to Trastuzumab, and led to reduced cytotoxicity response in HER2+ cancer cells treated with T-DM1.

**Conclusions:**

Our study provides novel evidence of Endo II function in HER2+ cancer cell motility and trafficking of HER2 that relates to effective treatments with trastuzumab or T-DM1. Thus, differential expression of Endo II may relate to sensitivity or resistance to trastuzumab-based therapies for HER2+ cancers.

**Electronic supplementary material:**

The online version of this article (doi:10.1186/s13058-017-0900-z) contains supplementary material, which is available to authorized users.

## Background

Mortality in patients with breast cancer is primarily due to the development of metastatic disease [1, 2]. This progression involves the acquisition of adaptive changes within tumor cells and the tumor microenvironment [3]. Gene expression profiling has identified four main subtypes of breast cancer that differ in driver genes and optimal therapies [4]. This classification has revealed different rates of breast cancer metastasis based on molecular subtype [5, 6]. The human epidermal growth factor receptor-2 (HER2)-positive (HER2+) subtype, characterized by overexpression of HER2 receptor, accounts for 20% of cases and has high rates of metastasis [7].

The monoclonal antibody trastuzumab (Herceptin™) has proven to be an effective adjuvant therapy against HER2+ breast cancers [8]. Trastuzumab treatment leads to reduced HER2 signaling, reduced tumor angiogenesis, cell cycle arrest, and enhanced anti-tumor immune responses [9]. Despite these beneficial outcomes, resistance to trastuzumab is common, and can occur early in the treatment regime [10]. Currently, the molecular mechanisms that explain HER2 suppression by trastuzumab remain unclear, with various hypotheses proposed [11–14]. However, there is a consensus that HER2-trastuzumab complexes are internalized by cells prior to loss of viability. Defining the underlying mechanisms of trastuzumab-induced HER2 internalization could provide insights into trastuzumab resistance.

Endophilin A2 (Endo II) is a ubiquitously expressed member of the BAR domain protein family [15]. Its structure consists of an N-terminal BAR domain that can sense and induce membrane curvature, and a C-terminal SH3 protein-protein interaction domain. Endo II is a scaffolding protein, shown to direct localization and subsequent activity of proteins involved in endocytosis and invadopodia [16, 17]. While the majority of studies have focused on its role in clathrin-mediated endocytosis (CME), recent evidence suggests Endo II can direct a distinct, clathrin-independent endocytic pathway known as fast endophilin-mediated endocytosis (FEME) [18, 19]. Furthermore, this novel process is found to take place at the leading edge of cells, and can regulate the ligand-induced internalization of tyrosine kinase receptors related to HER2 such as epidermal growth factor receptor (EGFR) [20]. These two characteristics imply that Endo II could play an important role in breast cancer progression, where an active leading edge and growth signaling promote cell motility and invasion. Previous studies have determined that Endo II plays an important role in cancer biology [17, 21]. We have previously reported that Endo II promotes growth, invasion, and metastasis in triple-negative breast cancer (TNBC) models [22]. There are currently no studies exploring the role of Endo II in HER2+ breast cancer, or its effects on response to treatment with trastuzumab.

In the current study, we profiled the expression of Endo II in HER2+ breast tumors and observed high expression in both tumors and lymph node metastases. In HER2+ cancer cells, Endo II silencing led to defects in HER2 internalization and signaling, and to impaired cell motility in vitro and tumor metastasis in vivo. Furthermore, Endo II silencing led to disruption of trastuzumab-induced HER2 internalization, cytotoxicity and cell motility in vitro.

## Methods

### Cell lines and antibodies

Normal-like MCF-10A and breast cancer cell lines (BT-474, SK-BR-3, MDA-MB-231, HCC1954) were obtained from American Type Culture Collection (ATCC). Cell lines were authenticated via STR profiling using the GenePrint® 10 System (Promega Corporation), which was performed at the Centre for Applied Genomics at the Hospital for Sick Children (Toronto, ON, Canada). Antibodies used in this study include: anti-Endophilin II (Santa Cruz Biotech (SCBT); H-60; rabbit), anti-RasGAP (rabbit) [23], anti–β-Actin (SCBT; C4; mouse), anti-EGFR (SCBT; 1005; rabbit), anti-phospho-EGFR (Cell Signaling Technologies (CST); pY1068; 1H12; mouse), anti-ERK1 (SCBT; K-23; rabbit), anti-phospho-ERK (SCBT; E-4; mouse), anti-Akt (CST; C67E7; rabbit), anti-phospho-Akt (CST; 193H12; rabbit), anti-HER2 (CST; 22425; rabbit), anti-phospho-HER2 (CST; 6B12; rabbit), anti-α-actinin (SCBT; H-300; rabbit), HRP-conjugated secondary antibodies (Licor; 926-80010 anti-mouse; 926-80011 anti-rabbit), FITC-conjugated anti-HER2 Affibody (Abcam; ab31889); PE-conjugated anti-HER2 (BioLegend; 24D2; mouse).

### Generation of stable Endo II KD cell lines and cell growth assays

Lentiviral pGIPZ and pLKO vectors were obtained for non-targeting (NT) short hairpin RNA (shRNA), empty vector control, and two Endo II-specific shRNAs (Open Biosystems). Lentiviral transduction of SK-BR-3 and HCC1954 cells followed by selection with puromycin was performed as previously described [22]. Endo II KD1 and KD2 cells in the GIPZ model correspond to clone V3LHS_345456 (target sequence 5′AGAACTGCTTCTTCAGCCC3′) and clone V3LHS_345454 (target sequence 5′TGACCTCGATGTCCAGGGA3′), respectively. Cell growth assays were performed as previously described [22].

### Transient overexpression of Endo II

The pEGFP-Endophilin II plasmid used to increase Endo II levels in BT-474 cells was provided by the Guan lab, and was previously described [17]. Cells were transfected using X-tremeGENE HP DNA Transfection Reagent (Roche). Immunogen complex was generated using a 4:1 ratio (4 μl reagent:1 μg DNA). Cells were harvested 48 hours after transfection for analysis of HER2 levels by flow cytometry as described subsequently.

### Cell lysis and immunoblotting

Cells lysis and immunoblot were performed as previously described [22]. Antibodies used and their dilutions include: Endo II (1:200); RasGAP (1:2,000); β-actin (1:2,000); EGFR (1:200); pEGFR (1:1,000); Erk (1:200); pErk (1:200); Akt (1:1,000); pAkt (1:1,000); HER2 (1:1000); pHER2 (1:1000); α-actinin (1:500); and HRP secondary anti-mouse and anti-rabbit (1:5000). Blots were imaged and analyzed on a C-Digit blot scanner (Licor) using Westernsure Premium ECL substrate (Licor) for phospho-antibodies, and Clarity ECL substrate (Bio-Rad) for pan-reactive antibodies.

### Surface HER2 analysis

To detect surface HER2 levels, cells were detached using 10 mM cold EDTA in PBS, and resuspended in cold 1% BSA PBS (10^6^ cells/ml). After 10 min of BSA blocking, 100 μl aliquots were incubated with 1 μl of FITC-conjugated or PE-conjugated HER2 antibodies for 30 min on ice) and washed three times with PBS prior to flow cytometry analysis (FC-500, Beckman-Coulter). Histograms were generated using FloJo 7 (FloJo, LLC). Experiments were also performed following treatment with either trastuzumab or EGF as detailed in the figure legends.

### Imaging of EGF uptake and HER2 internalization

For EGF uptake assays, HCC1954 pLKO cells and Endo KD cells seeded on coverslips were incubated with HER2 affibody for 30 min on ice, then switched to media containing Texas Red-conjugated EGF (100 ng/ml, Molecular Probes) at 37 °C for 15 min or kept on ice prior to fixation (1% paraformaldehyde for 15 minutes), and counterstaining with 4′,6-diamidino-2-phenylindole (DAPI) (1:400). Image acquisition was performed on a Quorum WaveFX-X1 spinning disc (Quorum Technologies Inc.), and a LSM800 Airyscan super-resolution confocal microscope (Zeiss). The images were analyzed for the presence and co-localization of EGF and HER2 marked vesicles using Image-Pro Plus 6 (Media Cybernetics).

### Cell migration and invasion assays

Cell migration and invasion assays were performed as previously described [22]. Briefly, HCC1954 and SK-BR-3 cells (5 × 10^4^) were seeded in Transwell inserts and allowed to migrate through 8-μm pores for 24 hours towards media with either EGF (50 ng/ml) or serum. For invasion assays, the Transwell filter was coated with Matrigel™ (BD; 50 μl of 20%). For kinetic studies of cell migration, HCC1954 GIPZ NT and Endo II KD cells were seeded on an ImageLock™ 96-well plate (25,000 cells/well). Wounds were made using WoundMaker™ (Essen Bioscience), rinsed and placed in an IncuCyte ZOOM with phase images taken every 2 hours for 48 hours (Essen Bioscience). The average wound density from triplicate wells was calculated using the instrument software.

### Tumor growth and lung seeding assays

Mammary orthotopic tumor growth and experimental metastasis assays were performed as previously described [22]. For mammary tumors, HCC1954 NT or Endo II KD cells (1.5 × 10^6^ in 50% Matrigel™) were used to inject mammary fat pads of Rag2^-/-^:IL2Rɣc^-/-^ mice, and tumor growth analyzed at 10 weeks. For lung seeding, HCC1954 NT or Endo II KD cells (8 × 10^5^ in 100 μl serum-free medium) were used to inject Rag2^-/-^:IL2Rɣc^-/-^ mice via the tail vein and lung metastases analyzed as previously described [22]. All procedures were approved by the Queen’s Animal Care Committee in accordance with Canadian Council on Animal Care guidelines.

### Cell viability and cytotoxicity assays

HCC1954 pLKO Vec and Endo II KD cells were seeded on a 96-well ImageLock™ plate (Essen BioScience), transfected with Nuclight Green BacMam 3.0 Reagent (Essen BioScience), and placed in an Incucyte ZOOM for imaging every 2 hours (Essen BioScience). After 24 hours, medium containing trastuzumab (84 μg/ml) and propidium iodide (PI, 1 μM; Biotium) was added and imaged for 48 hours. The number of Nuclight Green-positive cells in each field relative to the count at t = 0 was used to score cell growth. Cytotoxicity was calculated as PI-positive cells divided by total cells per field, then divided by t = 0. For trastuzumab emtansine (T-DM1) treatments, HCC1954 and SK-BR-3 NT and Endo II KD cells were treated with T-DM1 (50 ng/ml) and imaged as above. Quantification of relative cytotoxicity involved scoring PI+ cells relative to total cells marked by GFP expression, and divided by t = 0. For BT-474 cells, GFP-Endo II transfections were performed on day 1 as described above, and then seeded on a 96-well plate on day 2. Cells were treated with either TZ (84 μg/ml) or T-DM1 (50 ng/ml) in PI-containing medium on day 3 and imaged using an IncuCyte ZOOM instrument for 108 hours. GFP signal in transfected cells confirmed Endo II overexpression up until endpoint. Relative toxicity was measured by dividing the number of PI+ cells by the confluency of cells in the phase image relative to time zero. Endpoint cytotoxicity in HCC1954 GIPZ cells was measured using CytoTox Glo assay kit (Promega). Cells seeded on a 384-well plate (7500 cells/well), treated with vehicle (DMSO or PBS), trastuzumab, lapatinib (Toronto Research Chemicals), or paclitaxel (Cytoskeleton, Inc.) for 48 hours at the indicated doses. At endpoint, the relative toxicity consisted of the treatment sample luminescence relative to the untreated control.

### Immunohistochemical staining and scoring

Specificity of the Endo II antibody for immunohistochemical staining (IHC), and IHC scoring methods were described previously [22]. Tissue microarray (TMA) BR20837 containing primary breast tumors and lymph node metastases (US Biomax) and molecular subtyping information were used.

### Bioinformatic analysis

For analysis of Endo II expression in breast cancer microarray studies, a Kaplan–Meier (KM) curve for relapse-free survival for HER2+ node positive cases was created using Kaplan–Meier Plotter (www.kmplot.com) [24] based on high or low expression of *Sh3gl1* [22].

### Statistical analysis

Unless otherwise specified, all experiments were performed in triplicate and presented as mean ± SEM. H-scores from TMAs were analyzed using one-way analysis of variance (ANOVA). The unpaired Student’s two-tailed *t* test was used to compare control and knock-down (KD) cell lines, with significant differences defined by *P* < 0.05.

## Results

### High Endo II expression in metastatic HER2+ breast cancers and association with patient outcomes

**Fig. 1:**
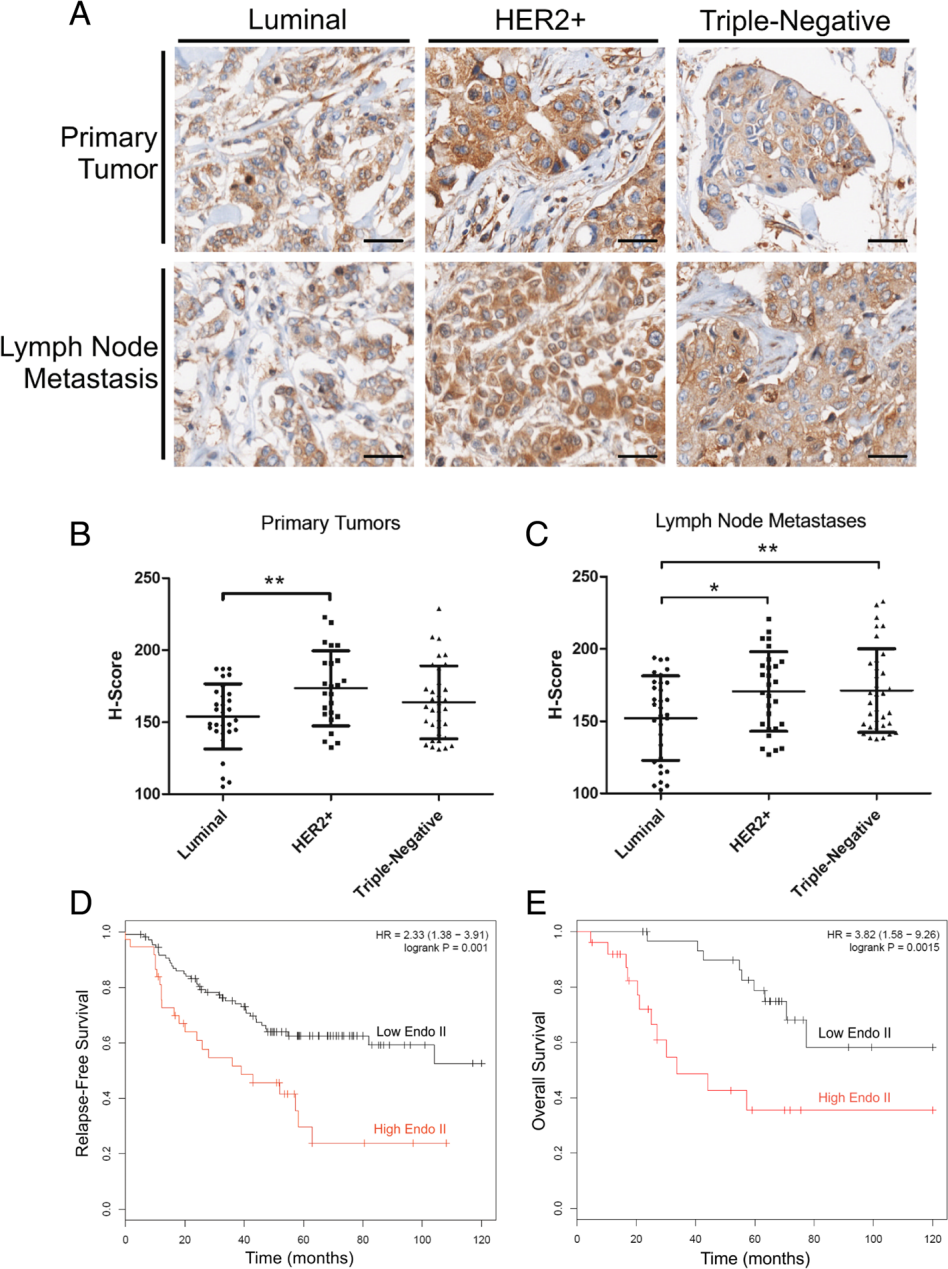
Endothilin A2 (Endo II) expression and association with poor prognosis in human epidermal growth factor receptor-2 (HER2)-positive (HER2+) breast cancer. **a** Representative images for immunohistochemical staining of Endo II in human breast tumors grouped by molecular subtype from a tissue microarray (TMA) with paired primary and lymph node metastases (n = 103). **b**, **c** Staining intensity was quantified using Imagescope software to generate tumor-specific H-scores for each primary tumor (**b**) or lymph node metastasis (**c**). **d**, **e** Kaplan–Meier plots for Endo II transcript levels (encoded by *Sh3gl1*) relative to relapse-free survival (**d**) (n = 146) and overall survival (**e**), (n = 56) are shown for patients with lymph-node positive HER2 tumors with up to 10 years of follow up. For high vs low Endo II groups, the median overall survival differences were 21 months (high Endo II) vs 63 months (low Endo II)

We previously reported high Endo II expression in TNBC tumors compared to luminal tumors, an association with poor prognosis, and a metastasis-promoting role for Endo II in TNBC xenograft models [22]. At that time, we observed a similar trend for the HER2+ subtype, but the number of HER2+ cases in that study cohort was limited. We performed additional IHC staining of Endo II in a larger TMA that included more HER2+ cases with matching primary tumors and lymph node samples. All primary tumor and lymph node samples expressed Endo II, with the highest levels observed in HER2+ cases (Fig. 1a). Quantification of the tumor-specific Endo II staining was performed with imaging software to generate H-scores [25], confirming that HER2+ primary tumors had significantly higher Endo II expression relative to luminal tumors (Fig. 1b). In this cohort, Endo II expression was similar in HER2+ and TNBC tumors (Fig. 1b). Similar results were observed in lymph node metastases, with Endo II levels in both HER2+ and TNBC metastases significantly higher than in luminal cases (Fig. 1c). Although patient outcomes were not available for the cohort previously mentioned, we tested whether differential expression of Endo II gene *Sh3gl1* had any significant associations with outcomes in patients with node-positive HER2+ breast cancer using open-access Kaplan–Meier Plotter microarray data [26]. It is worth noting that this cohort predated the development of targeted therapies for HER2+ cancers. Relapse-free survival was significantly longer in patients with low *Sh3gl1* expression compared to those with high expression (Fig. 1d). Similar results were observed for overall survival in these patients (Fig. 1e), which corresponded to a mean survival time of 63 months in the low Endo II cohort, compared to 21 months in the high Endo II cohort. High Endo II expression also correlated with reduced metastasis-free survival rates and reduced relapse-free survival in chemotherapy-treated patients in this cohort (Additional file 1: Figure S1a and b). Similar correlations were observed in relapse-free survival among node-negative patients, and when eliminating stratification by node status (Additional file 1: Figure S1c and d). We also extended this analysis to lamellipodin, a binding partner of Endo II that functions in FEME, and found that high lamellipodin transcript levels (encoded by *RAPH1*) was associated with significantly worse relapse-free survival in node positive HER2+ patients (Additional file 1: Figure S1e). Together, these results show that Endo II is highly expressed in a subset of HER2 breast cancers and may be associated with poor clinical outcomes.

### Increased HER2 levels upon Endo II silencing in HER2+ breast cancer cells

**Fig. 2:**
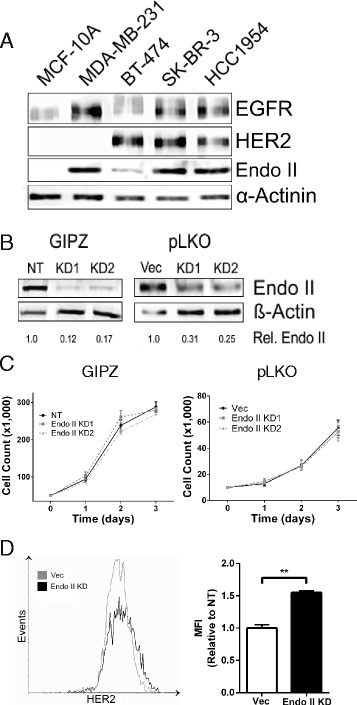
Elevated surface human epidermal growth factor receptor 2 (HER2) levels upon Endo II silencing in HER2-positive human breast cancer cells. **a** Lysates from a panel of human breast cell lines were subjected to immunoblot with antibodies to epidermal growth factor receptor (EGFR), HER2, endothelin A2 (Endo II), and loading control α-Actinin. **b** Immunoblot analysis of HCC1954 cells with expression of two different shRNAs from GIPZ or pLKO lentiviral vectors resulted in stable knock-down (KD) of Endo II. Controls included cell lines expressing either a non-targeting shRNA (NT) or an empty vector (Vec) for GIPZ and pLKO models, respectively. **c** Cell growth curves were prepared by counting viable HCC1954 control and Endo II KD cells for both GIPZ (left) and pLKO (right) models at 24, 48 and 72 hours post plating. **d** Flow cytometry analysis of surface HER2 levels on HCC1954 pLKO Vec and Endo II KD1 cells measured using a HER2 affibody, and displayed as a representative histogram (left) or by the median fluorescence intensity (MFI) for three independent experiments (**P < 0.01)

To directly study the role of Endo II in human HER2+ breast cancer cell lines, we first profiled Endo II expression in two HER2+ cell lines (SK-BR-3, HCC1954) alongside lines representing TNBC and luminal subtypes (MDA-MB-231 and BT-474, respectively), and a normal-like breast epithelial cell line (MCF-10A). We observed higher Endo II levels in TNBC and HER2+ cancer cell lines, including SK-BR-3 and HCC1954 cells, which co-expressed EGFR and HER2 (Fig. 2a). We selected these two cell lines for stable shRNA-mediated KD of Endo II, or for the expression of an NT control shRNA by lentiviral transduction using pGIPZ vectors. Compared to NT controls, we achieved ~85% KD in Endo II levels with two separate shRNAs (KD1, KD2) in both HCC1954 cells (Fig. 2b), and in SK-BR-3 cells (Additional file 1: Figure S2a). We also achieved Endo II KD using another lentiviral shRNA vector (pLKO) that lacked the GFP reporter encoded in the GIPZ vector to facilitate imaging studies, and achieved a similar Endo II KD efficiency (Fig. 2b, right panels, ~70–75% KD). No significant changes in cell growth rates with stable Endo II KD were observed for these HER2+ cell lines with either lentiviral vector system (Fig. 2c, Additional file 1: Figure S2b). However, flow cytometry analysis revealed significantly increased levels of HER2 on the cell surface of Endo II KD cells compared to vector control HCC1954 cells (Fig. 2d). Furthermore, transient overexpression of GFP-Endo II in BT-474 cells, with low endogenous Endo II expression, led to reduced levels of HER2 on the cell surface (Additional file 1: Figure S3). Together, these results implicate Endo II in promoting HER2 internalization in HER2+ cancer cells.

### Endo II silencing impairs HER2 internalization and downstream signaling

**Fig. 3:**
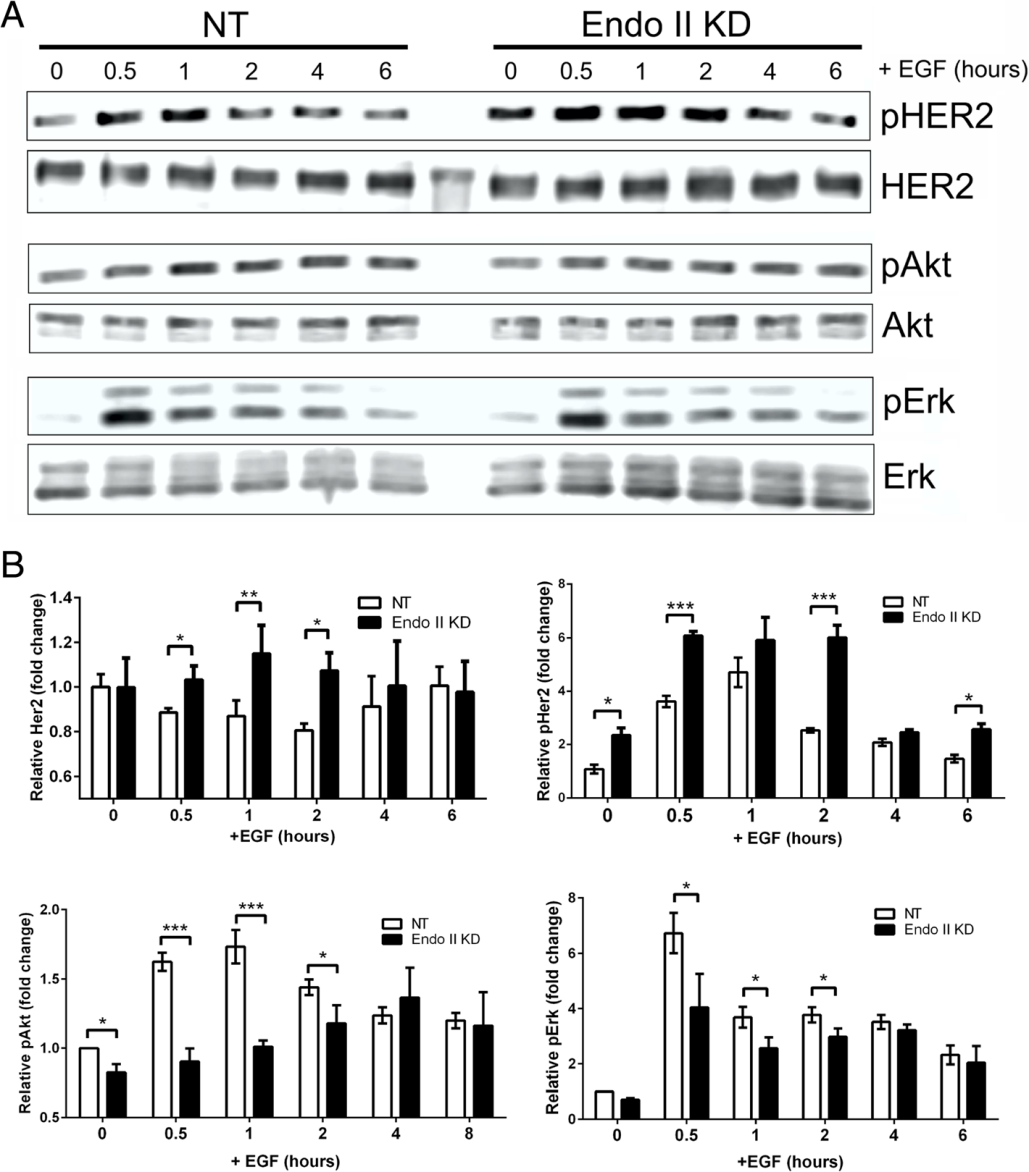
Impaired human epidermal growth factor receptor 2 (HER2) signaling upon endothelin A2 (Endo II) silencing in HER2-positive cancer cells. **a** Serum-starved HCC1954 GIPZ non-targeting (NT) and Endo II knock-down (KD)1 cells were treated with or without epidermal growth factor (EGF) (100 ng/ml) for up to 6 hours and subjected to immunoblot with antibodies to the indicated proteins. **b** Graphs depict the results of densitometry analysis of the EGF treatment effects on HER2 levels and phosphorylation state indicated by the ratio of phosho-specific relative to pan-reactive antibodies for the indicated proteins from three independent experiments (*P < 0.05, **P < 0.01, ***P < 0.001)

With our findings that Endo II regulates HER2 levels, we reasoned that Endo II may also regulate HER2 signaling. Since we had already linked Endo II to EGFR signaling in TNBC cells [22], we selected SK-BR-3 and HCC1954 cells that highly express both EGFR and HER2 to study the effects of Endo II KD on EGF-induced EGFR and HER2 signaling. In SK-BR-3 cells that were serum-starved and treated with EGF (0–16 minutes), we observed similar levels of EGFR phosphorylation, and a trend towards reduced downstream phosphorylation of Akt and Erk kinases with Endo II silencing (Additional file 1: Figure S4). In HCC1954 cells treated with EGF for up to 6 hours, Endo II KD cells retained higher levels of HER2 and phospho-HER2 (pHER2) compared to NT control cells (Fig. 3a; densitometry data in Fig. 3b). The reduced levels of HER2 in NT cells after 2 hours of EGF treatment may be due to ubiquitylation, internalization and degradation of HER2 in these cells. Indeed, we were able to detect ubiquitylated forms of HER2 in EGF-treated NT control cells, but not in Endo II KD cells in preliminary studies (data not shown). In addition, the kinetics and extent of EGF-induced signaling to Akt and Erk kinases was significantly lower in Endo II KD cells compared to control (Fig. 3a and b). Together, these results suggest that Endo II regulates HER2 downregulation and downstream signaling upon HER2 activation.

**Fig. 4:**
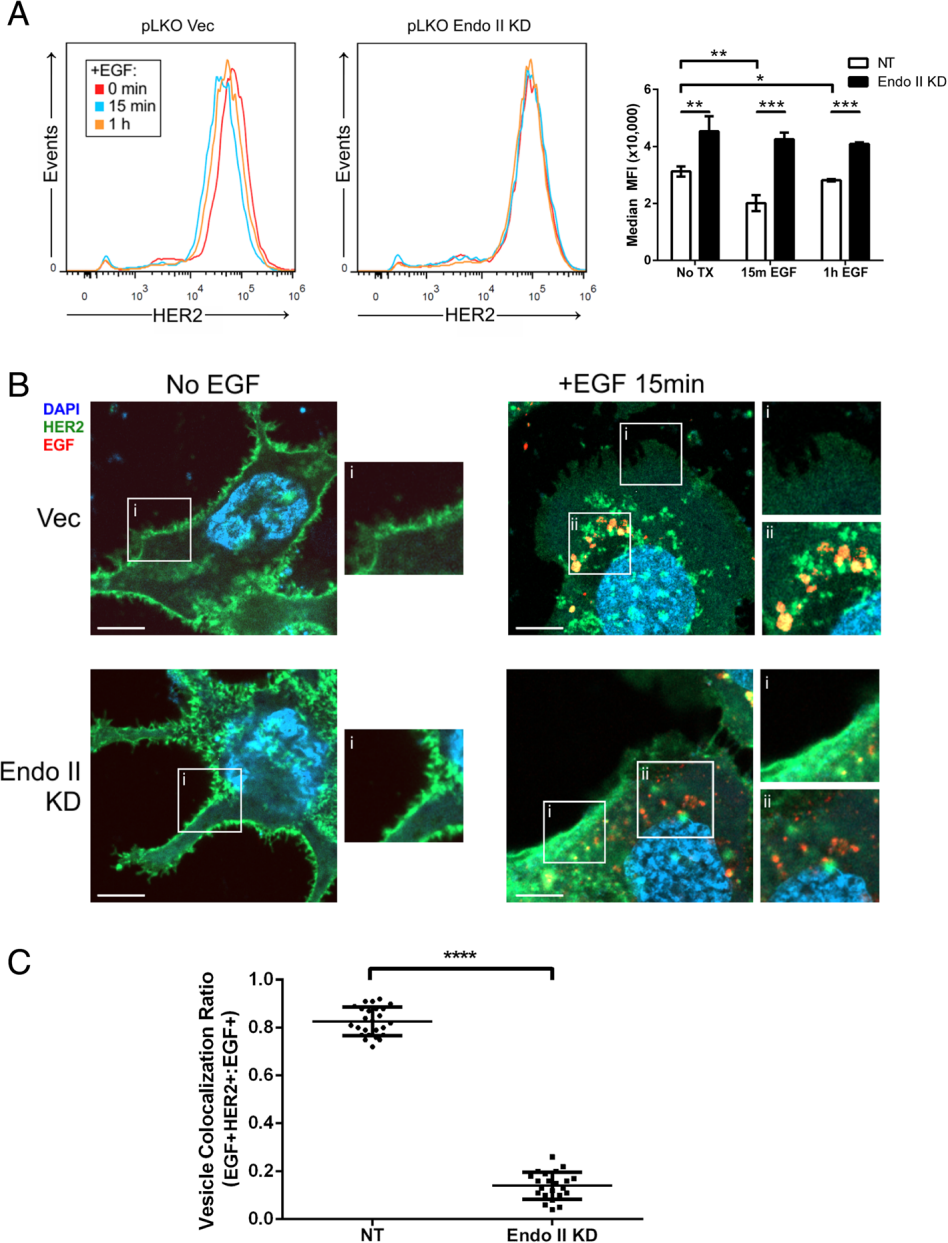
Endothelin A2 (Endo II) promotes human epidermal growth factor receptor 2 (HER2) internalization in response to epidermal growth factor (EGF) treatment of HER2-positive cancer cells. **a** Flow cytometry analysis of surface HER2 levels in HCC1954 pLKO vector (Vec) and Endo II knock-down (KD)1 cells treated with or without EGF (100 ng/ml) for 15 minutes (min) or 1 hour (h) and stained with HER2 affibody. A representative histogram (left) and mean fluorescence intensity (MFI) results from three experiments are shown (*P < 0.05, **P < 0.01, ***P < 0.001). **b** Representative confocal microscopy images are shown for HCC1954 pLKO Vec and Endo II KD1 cells treated with or without Texas Red-EGF (100 ng/ml) and HER2 affibody (green; DAPI counterstain also shown). Inserts were selected to highlight membrane (i) and internalized vesicle (ii) compartments. **c** Quantification of vesicles containing co-localized HER2 and EGF in HCC1954 pLKO Vec and Endo II KD1 cells treated for 15 minutes from 25 cells (results are representative of two independent experiments)

To directly measure the effects of Endo II silencing on the rate of HER2 internalization, the surface levels of HER2 were monitored by affibody staining of HER2 during EGF treatment of cells and analyzed by flow cytometry. As expected, EGF treatment for 15 minutes led to HER2 internalization, marked by a left shift in the histogram and reduced mean fluorescence intensity (MFI) of HER2 staining in control HCC1954 pLKO cells (Fig. 4a and b). The partial recovery of HER2 levels following 1 hour of EGF treatment suggested that a recycling pathway was engaged in HCC1954 control cells (Fig. 4a and b). Although the surface levels of HER2 were higher at baseline in Endo II KD cells, surface HER2 levels remained unchanged following EGF treatment (Fig. 4a and b). Similar results were observed with independent reagents for HER2 immunostaining and Endo II KD in our HCC1954 GIPZ model (Additional file 1: Figure S5). To directly visualize HER2 localization, HCC1954 pLKO and Endo II KD cells were treated with Alexa488-conjugated HER2 affibody with or without treatment with Texas Red-labeled EGF (TR-EGF). Following fixation and staining of nuclei with DAPI, confocal microscopy was performed. As expected, HER2 localized to the cell periphery in the absence of TR-EGF treatment in both vector control and Endo II KD cells (Fig. 4b). Upon treatment with TR-EGF for 15 minutes, HER2 mostly co-localized with TR-EGF within endosomes in vector control cells, but this was less apparent in Endo II KD cells (Fig. 4b). This was despite the presence of EGF-positive vesicles in Endo II KD cells, where a significantly reduced fraction of these vesicles were also positive for HER2 compared to control cells (Fig. 4c). Together, these results implicate Endo II in HER2 internalization and downstream signaling in HER2+ cancer cells in vitro.

### Endo II promotes HER2+ cancer cell motility, invasion and metastasis

**Fig. 5:**
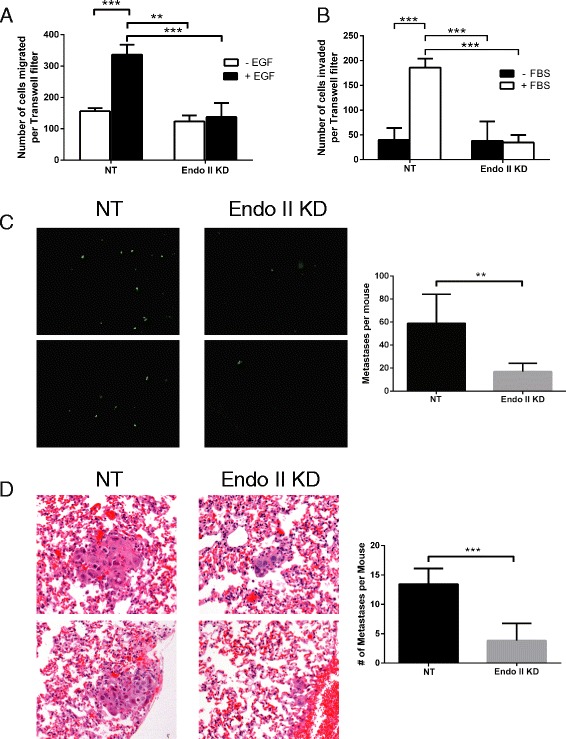
Endothelin A2 (Endo II) promotes human epidermal growth factor receptor 2 (HER2)-positive cancer cell motility and tumor metastasis. **a** HCC1954 pGIPZ non-targeted (NT) and Endo II knock-down (KD)1 cells were seeded in Transwell inserts (8 μm pores) and allowed to migrate towards the lower chamber containing serum-free medium supplemented with or without epidermal growth factor (EGF) (50 ng/ml) for 24 hours. Graph depicts the average cells per filter from assay triplicates and from three independent experiments (mean ± SEM; **P < 0.01, ***P < 0.001). **b** HCC1954 pGIPZ NT and Endo II KD1 cells were seeded in Transwell inserts coated with Matrigel and allowed to invade through the matrix and filter towards medium supplemented with 10% serum. Graph depicts the average cells per filter from assay triplicates and from three independent experiments (mean ± SEM; ***P < 0.001). **c**, **d** Experimental metastasis assays were conducted by tail vein injection of HCC1954 pGIPZ NT or Endo II KD1 cells (8.0 × 10^5^) and after 2 weeks, GFP+ lung metastases were visualized and scored in mouse lung tissue (**c**) and from H&E-stained lung tissue sections (**d**) (*n* = 8/group, from two separate experiments; **P < 0.01, ***P < 0.001)

Next, we studied the effects of Endo II silencing on the motile and invasive properties of HER2+ cancer cells. Using Transwell filter assays, chemotaxis of HCC1954 NT and KD cells towards a gradient of EGF was measured. Compared to NT control cells that showed the expected increase in motility in response to EGF, Endo II KD cells showed significantly less chemotaxis response (Fig. 5a). This defect in motility with Endo II KD was also observed in SK-BR-3 cells, both in response to serum and EGF (Additional file 1: Figure S6). It is worth noting that similar results were observed with two separate shRNAs, which is consistent with Endo II silencing effects, not off-target effects. Furthermore, Endo II silencing also impaired the ability of HCC1954 cells to invade through Matrigel-coated Transwell filters in response to serum (Fig. 5b).

To study the effects of Endo II on HER2+ tumor growth and metastasis in vivo, we performed both mammary orthotopic xenograft assays and experimental metastasis assays in mice lacking natural killer (NK) cells, B cells and T cells (Rag2^-/-^:IL2Rγc^-/-^). Following the injection of HCC1954 NT and Endo II KD cells (2 × 10^6^) in the mammary fat pad, tumor growth was monitored for 10–11 weeks. During this period and at endpoint, we observed no significant differences in tumor growth with Endo II silencing (Additional file 1: Figure S7). We also failed to detect sufficient metastatic nodules to score the effects of Endo II on spontaneously arising metastases (data not shown). However, using experimental metastasis assays that measured lung seeding efficiency following tail vein injections of HCC1954 NT and Endo II KD cells (8 × 10^5^), GFP+ lung metastases were observed after 14 days (Fig. 5c). Quantification of these metastases using the GFP signal showed a significant reduction in metastatic nodules with Endo II KD (Fig. 5c). Histological staining of lung tissue sections was also used to score metastases, which were significantly higher for NT control cells compared to Endo II KD (Fig. 5d). Together, these results identify Endo II as a positive regulator of HER2+ cancer cell invasion and tumor metastasis.

### Endo II regulates trastuzumab-induced HER2 internalization and blockade of HER2+ cancer cell motility

**Fig. 6:**
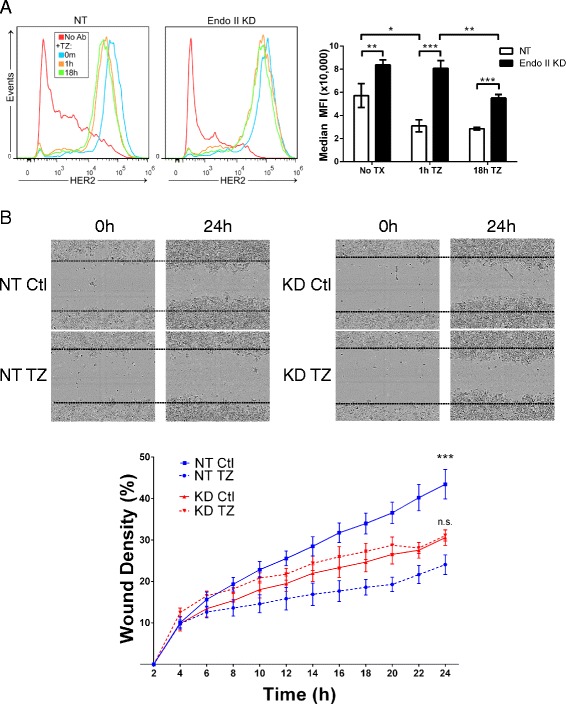
Endothelin A2 (Endo II) promotes trastuzumab-induced human epidermal growth factor receptor 2 (HER2) internalization and suppression of HER2-positive cancer cell motility. **a** Flow cytometry analysis was used to measure effects of trastuzumab (TZ, 84 μg/ml) on HER2 levels in HCC1954 pGIPZ (NT) or Endo II knock-down (KD)1 cells treated for up to 18 hours and stained with phycoerythrin (PE)-anti-HER2. Representative histograms (left) and a graph of changes in mean fluorescent intensity (MFI) of HER2 staining from three experiments are shown (*P < 0.05, **P < 0.01, ***P < 0.001). **b** HCC1954 pGIPZ NT and Endo II KD cells seeded in a 96-well plate and after 18 hours wound areas were generated in the cell monolayers prior to treatment with or without trastuzumab (84 μg/ml). Following real-time imaging for 24 hours, the extent of cell migration into the wound area was visualized (left, representative images shown) and quantified from triplicate wells (right) for NT controls (upper; **significant difference at endpoint with TZ treatment) and Endo II KD cells (lower; no significant differences at endpoint were observed with TZ treatment)

The antibody trastuzumab (TZ, Herceptin™) inhibits HER2+ tumor growth, and since approval for clinical use, has greatly improved outcomes for HER2+ patients with cancer [8, 27]. Since the mode of action of TZ has been suggested to include HER2 internalization and degradation [11], we investigated whether Endo II plays a role in TZ response of HER2+ breast cancer cells. HCC1954 NT and Endo II KD cells were treated with or without TZ and the surface levels of HER2 measured by flow cytometry. In NT control cells, TZ treatment led to a significant decrease in surface HER2 levels within 1 hour, and remained lower for 18 hours of treatment (Fig. 6a). In contrast, HER2 levels remained higher on the cell surface of TZ-treated Endo II KD cells throughout the time course, with a more modest decrease at the 18-hour mark (Fig. 6a; see graph on the right for quantification). We observed very similar results in the HCC1954 pLKO Endo II KD cell model (Additional file 1: Figure S8a).

To test the effects of TZ treatment on HER2 phosphorylation and degradation, HCC1954 NT and Endo II KD cells were treated with TZ for up to 8 hours. Immunoblot analysis revealed a rapid decrease in HER2 phosphorylation (pHER2) that preceded downregulation of HER2 in NT control cells (Additional file 1: Figure S8b and c). In contrast, Endo II KD cells showed a delayed response to TZ-induced suppression of pHER2 and HER2 protein levels (Additional file 1: Figure S8 and c). These results identify a role for Endo II in promoting HER2 internalization and degradation upon treatment of HER2+ cancer cells with TZ.

Since HER2 signaling also promotes cell migration, we performed a time lapse wound healing assay over 24 hours on HCC1954 NT and Endo II KD cells in the presence and absence of TZ. In control cells, TZ treatment led to a significant reduction in wound healing migration (Fig. 6b). In contrast, Endo II KD cells showed reduced motility, and no further inhibition with TZ was observed (Fig. 6b). Together, these results implicate Endo II in mediating efficient TZ responses in HER2+ cancer cells, including HER2 internalization, degradation, and suppression of cell motility.

### Endo II promotes sensitivity to TZ and T-DM1 treatment in HER2+ cancer cells

**Fig. 7:**
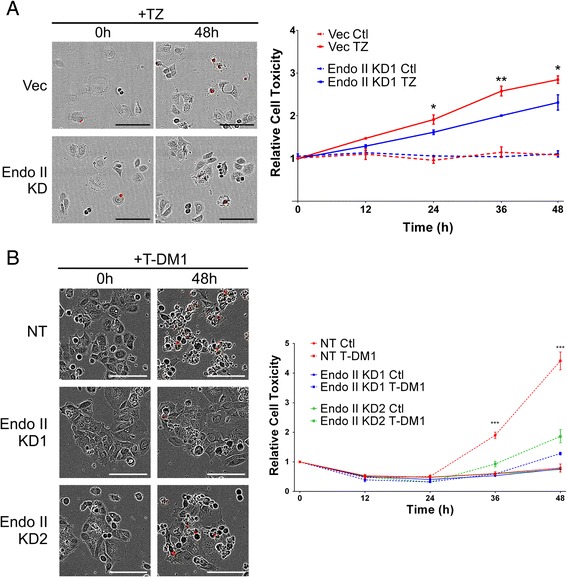
Endothelin A2 (Endo II) promotes the cytotoxicity of trastuzumab and T-DM1 in human epidermal growth factor receptor 2-positive (HER2+) cancer cells. **a** HCC1954 pLKO vector (Vec) and Endo II knock-down (KD)1 cells seeded in a 96-well plate were transfected with Nuclight2 live-cell nuclear stain, and treated with or without trastuzumab (TZ, 84 μg/ml) in medium supplemented with propidium iodide (PI; compared to control (ctl) with no drug added). Following real-time imaging, the relative increase in cytotoxicity (PI+ cells relative to Nuclight2+ cells) was analyzed over 48 hours for three experiments performed in triplicate. Representative merged images of red channel (PI) and bright field are shown on the left. The graph on the right indicate mean values (± SEM) from three experiments (significant differences between Vec and Endo II KD cells treated with TZ are denoted by *P < 0.05, **P < 0.01). **b** HCC1954 NT, Endo II KD1 and KD2 cells seeded in a 96-well plate were treated with or without trastuzumab emtansine (T-DM1) (50 ng/ml) in medium supplemented with PI compared to untreated control (Ctl). Following real-time imaging, the relative cell toxicity (calculated as PI+ cells relative to GFP+ cells) was analyzed over 48 hours for three experiments performed in triplicate. Representative merged images of red channel (PI) and bright field are shown on the left. The graph on the right indicates mean values (± SEM) from three experiments (significant differences between non-targeted (NT) and Endo II KD cells treated with T-DM1 are denoted by ***P < 0.001)

Next, we tested the effects of Endo II on TZ and the antibody drug conjugate T-DM1 on killing of HER2+ breast cancer cells. HCC1954 vector and Endo II KD cells were transfected with Nuclight™ live-cell nuclear stain, then treated with TZ in medium containing propidium iodide (PI) and fluorescence and bright-field images were acquired every 2 hours for 48 hours. As expected, TZ treatment led to increased cytotoxicity in vector control cells between 24 and 48 hours, but this effect was significantly reduced in Endo II KD cells (Fig. 7a; results shown from three independent experiments). Similar results were also obtained using a luciferase-based cytotoxicity assay performed on HCC1954 NT and Endo KD cells (Additional file 1: Figure S9a). Interestingly, when cells were treated with the EGFR/HER2 small molecule inhibitor lapatinib, which targets both intracellular and surface pools of EGFR and HER2, we observed no difference in cytotoxicity with Endo II KD (Additional file 1: Figure S9b). Likewise, the chemotherapy drug paclitaxel was equipotent on both NT control and Endo II KD cells (Additional file 1: Figure S9c). Together, these results suggest that Endo II is a key factor in achieving a favorable response to TZ in HER2+ breast cancer cells, but not for small molecule HER2 inhibitors or chemotherapy responses in these cells.

Since T-DM1 is emerging as an important new line of therapy for metastatic HER2+ cancers, and depends on HER2 internalization for release of the cytotoxic payload emtansine [28], we next tested the role of Endo II in T-DM1 treatment response. Using the PI assay described above, we tested T-DM1 treatment effects on the viability of HCC1954 NT, Endo II KD1, and Endo II KD2 cells over 48 hours. At much lower doses than that used for TZ, we observed a significant increase in cytotoxicity (PI+ cells) in NT control cells treated with T-DM1 for 36–48 hours of treatment (Fig. 7b). In contrast, the cytotoxicity of T-DM1 was significantly impaired in both Endo II KD cell lines (Fig. 7b). We observed similar results with Endo II silencing in SK-BR-3 cells, with reduced cytotoxicity upon treatment with T-DM1 compared to control (Additional file 1: Figure S9d). Furthermore, BT-474 cells transiently transfected with a GFP-Endo II construct showed increased sensitivity to T-DM1 treatment compared to a mock transfection control (Additional file 1: Figure S10). Taken together, these results implicate Endo II in promoting HER2 endocytosis and delivery of the cytotoxic payload of clinical grade antibody drug conjugate T-DM1 in HER2+ cancer cells.

## Discussion

Recent studies implicate the endocytic adaptor protein Endo II in regulating EGFR signaling, invadopodia formation, trafficking of matrix metalloproteinase MT1-MMP, cancer stemness and metastasis [17, 20–22]. To evaluate the relevance of Endo II in HER2+ cancers that are dependent on HER2 signaling [29], we tested the effects of Endo II silencing on HER2+ cancers and their response to HER2-targeted therapies. Here we reported that HER2+ tumors express Endo II at high levels, and high Endo II transcript levels are associated with poor prognosis in node-positive cases. We also showed that Endo II silencing leads to impaired HER2 internalization and degradation in response to EGF-induced clustering with EGFR. This correlated with defects in signaling, with impaired HER2 cancer cell motility, and with reduced tumor metastasis in vivo. Silencing of Endo II in HER2+ cancers also limited the response of these cells to trastuzumab, with observed defects in HER2 internalization, cytotoxicity, and cell migration. Last, we showed that Endo II promotes killing of HER2+ cancer cells treated with the clinical-grade antibody drug conjugate T-DM1. Overall, these findings implicate Endo II in HER2 endocytosis, signaling, and HER2+ cancer cell invasion, and in effective HER2 pathway blockade by trastuzumab and T-DM1. Future studies of Endo II and the endocytic pathways in which it participates may be of particular relevance to predicting the response of HER2+ tumors to treatment with trastuzumab or T-DM1.

This study identified Endo II as a key regulator of HER2 internalization and signaling in HER2+ breast cancer cells. Interestingly, Endo II silencing produced a similar phenotype in EGFR trafficking and signaling in TNBC models [22]. Together, these studies broaden our understanding of HER internalization and trafficking via Endo II-driven mechanisms such as FEME. It will be important to further test whether Endo II and FEME are also important regulators of the other HER family receptors that heterodimerize with HER2. Indeed, HER3 is a known negative prognostic indicator in HER2+ breast cancers [30]. The current lack of robust and validated assays for HER3 and HER4 expression impedes accurate prediction of patient response to HER-family targeted therapy [31]. To maximize the efficacy of these treatments, it will also be important to define the sensitivity of these co-receptors to HER2 inhibitors.

The effects of Endo II silencing on HER2 signaling may relate to the defects in HER2 internalization reported here, and highlight the importance of compartmentalized signaling to downstream pathways by receptor tyrosine kinases within endosomes [32, 33]. However, HER2 can also signal to downstream pathways while on the cell surface [34]. Since the rate of EGFR-HER2 heterodimer internalization is delayed compared to that of EGFR homodimers [34], this may explain how EGF uptake was still observed in Endo II KD HER2+ cells. It is also possible that Endo II silencing may shift the route of HER internalization towards clathrin-mediated endocytosis (CME), or other non-clathrin endocytosis pathways [35, 36], which will require further investigation.

This study implicates Endo II in promoting the invasiveness and metastatic potential of HER2+ cancer cells. Previous studies including our own have identified Endo II as a key regulator of matrix metalloproteinase MT1-MMP (MMP-14) internalization and trafficking [17, 22]. MT1-MMP localizes to invadopodia, which are filamentous actin-rich projections that degrade the extracellular matrix (ECM) and promote cancer cell invasion [37, 38]. In TNBC cells, Endo II silencing causes defects in invadopodia maturation, ECM degradation, invasion and metastasis [22]. We predict that Endo II likely facilitates invadopodia in HER2+ cancers by facilitating the recycling of MT1-MMP and HER2 at the leading edge of these highly invasive cancer cells.

Here we also showed that Endo II regulates HER2 internalization induced by trastuzumab in HER2+ cancer cells. As a frontline therapy for HER2+ breast cancer, trastuzumab has dramatically improved outcomes for most patients [8], but not all patients have a favorable response and many develop resistance [39]. Thus, a deeper understanding of the molecular mechanisms behind HER2 blockade by trastuzumab may help develop strategies to avoid resistance and improve response rates. Some research suggests that trastuzumab does not direct internalization of HER2, but may impact HER2 trafficking at later steps [11]. It has also been shown that caveolin-1 promotes internalization of the antibody drug conjugate T-DM1 through caveolae [40]. Our findings implicate Endo II and Endo II-dependent endocytosis in the internalization and downregulation of HER2 in HER2+ cancers treated with trastuzumab. Further studies will be required to test if altered Endo II endocytic processes may explain resistance to these antibody-based treatments in HER2+ cells.

This study also implicates Endo II in regulating trastuzumab-induced cytotoxicity in HER2+ cancer cells. Indeed, G1 cell cycle arrest due to p27^Kip1^ suppression of CDK2 activity is a documented effect of trastuzumab treatment [41]. Blockade of HER2 can also limit expression of the anti-apoptotic Mcl-1 protein, and promote apoptosis [42, 43]. These studies should be expanded to relevant HER2+ tumor models in vivo since trastuzumab also promotes antibody-directed cell cytotoxicity [44–46]. Given the potent effects of HER2 on cancer cell motility and tumor metastasis [47, 48], it will also be important to measure the effects of Endo II silencing on metastasis in these tumor models following trastuzumab treatment.

The identification of HER2 as a biomarker and clinical target in a subset of breast cancers has led to significant improvements in therapy and outcomes for patients [8, 49]. Despite the success of trastuzumab as an adjuvant treatment, resistance to this therapy is frequently observed [9, 39, 50]. Thus, future validation of biomarkers that predict treatment response or relapse probability should be prioritized. Given our findings here that implicate Endo II in promoting trastuzumab response, but not that of lapatinib, it would be interesting to profile Endo II levels in tumor biopsies from the MA.31 clinical trial that compared trastuzumab and lapatinib for treatment of HER2+ patients with advanced breast cancer [51]. If high or low Endo II expression correlates with sensitivity to either targeted therapy, it would support further testing as a predictive biomarker for HER2-targeted therapies in larger cohorts. Furthermore, since T-DM1 responses rely on HER2 internalization [28], and we show here that Endo II promotes T-DM1-induced cytotoxicity, further studies of Endo II-mediated molecular mechanisms may inform how to maximize the delivery of the T-DM1 cytotoxic warhead and other antibody drug conjugates. The findings in the current study suggest that endocytic pathways that may become deregulated in cancer are highly relevant to the successful delivery of antibody-based cancer therapies.

## Conclusions

This study provides novel evidence that Endo II promotes invasive phenotypes in HER2-driven breast cancers, and that high Endo II levels are linked to poor prognosis in a cohort of HER2+ cancer patients. However, it is worth noting that this cohort of patients was treated prior to the discovery and clinical approval of trastuzumab or T-DM1. With regard to these targeted therapies, Endo II promotes both HER2 internalization and the killing of HER2+ cancer cells by the targeted therapies trastuzumab and T-DM1. While further studies in larger cohorts are required, our results suggest that tumors with high Endo II expression may predict a favorable response to trastuzumab or T-DM1 and have high risk for progression to metastatic disease.

## Additional file

Additional file 1: Figure S1. Endo II expression and association with risk of metastasis and chemotherapy response in HER2+ breast cancers. **Figure S2.** Endo II silencing in SK-BR-3 cells. **Figure S3.** Endo II overexpression leads to reduced surface HER2. **Figure S4.** Endo II promotes EGF-induced Akt activation in SK-BR-3 cells. **Figure S5.** Endo II promotes HER2 internalization in HCC1954 cells. **Figure S6.** Endo II promotes SK-BR-3 cell motility. **Figure S7.** Endo II silencing has no overt effects on HER2+ tumor growth. **Figure S8.** Endo II promotes HER2 internalization and downregulation following trastuzumab treatment of HCC1954 cells. **Figure S9.** Endo II promotes cytoxicity upon treatment with trastuzumab, but not with lapatinib, in HER2+ cancer cells. **Figure S10.** Endo II overexpression results in increased sensitivity to T-DM1. (DOCX 1965 kb)

## Abbreviations

BAR: Bin1/Amphiphysin/Rvs
DAPI: 4′,6-Diamidino-2-phenylindole
ECM: Extracellular matrix
EGF: Epidermal growth factor
EGFR: Epidermal growth factor receptor
Endo II: Endophilin A2
FEME: Fast endophilin-mediated endocytosis
FITC: Fluorescein isothiocyanate
GFP: Green fluorescent protein
HER2: Human epidermal growth factor receptor-2
HER2+: HER2 positive
HRP: Horseradish peroxidase
IHC: Immunohistochemical staining
KD: Knock-down
KM: Kaplan–Meier
NK: Natural killer
NT: Non-targeting
PBS: Phosphate-buffered saline
PE: Phycoerythrin
PI: Propidium iodide
SH3: Src homology 3
STR: Short tandem repeat
T-DM1: Trastuzumab emtansine
TMA: Tissue microarray
TNBC: Triple-negative breast cancer
TZ: Trastuzumab
