# Supplementary material for: Endophilin A2 promotes HER2 internalization and sensitivity to trastuzumab-based therapy in HER2-positive breast cancers
Source: Breast Cancer Res. 2017 Oct 3;19:110. doi: 10.1186/s13058-017-0900-z (PMC5627411; doi:10.1186/s13058-017-0900-z)
Supplement: Additional file 1: Figure S1. — Endo II expression and association with risk of metastasis and chemotherapy response in HER2+ breast cancers. Figure S2. Endo II silencing in SK-BR-3 cells. Figure S3. Endo II overexpression leads to reduced surface HER2. Figure S4. Endo II promotes EGF-induced Akt activation in SK-BR-3 cells. Figure S5. Endo II promotes HER2 internalization in HCC1954 cells. Figure S6. Endo II promotes SK-BR-3 cell motility. Figure S7. Endo II silencing has no overt effects on HER2+ tumor growth. Figure S8. Endo II promotes HER2 internalization and downregulation following trastuzumab treatment of HCC1954 cells. Figure S9. Endo II promotes cytoxicity upon treatment with trastuzumab, but not with lapatinib, in HER2+ cancer cells. Figure S10. Endo II overexpression results in increased sensitivity to T-DM1. (DOCX 1965 kb) [file 13058_2017_900_MOESM1_ESM.docx]

**
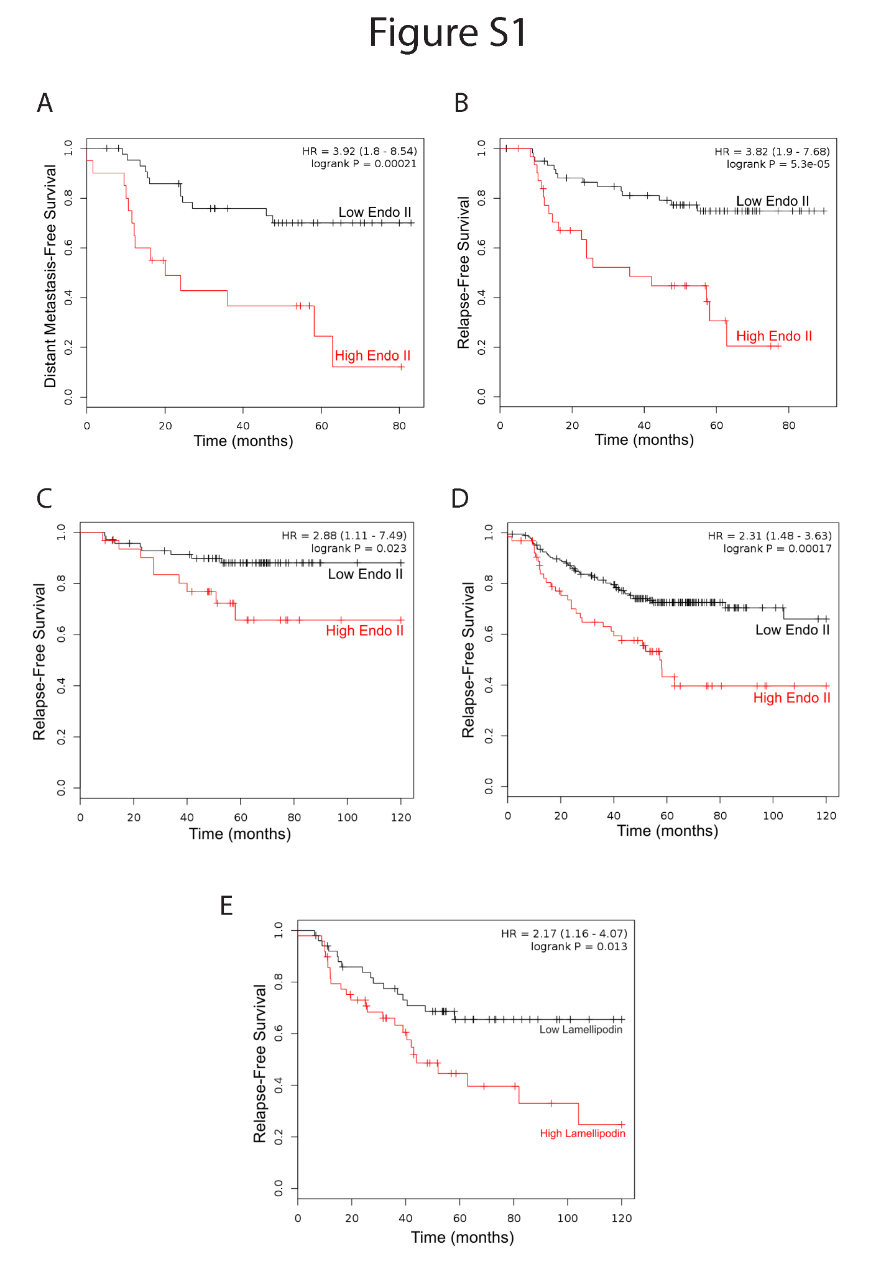
**

**Figure S1** **Endo II expression and association with risk of metastasis and chemotherapy response in HER2+ breast cancers**. **(A)** Kaplan-Meier plot for high or low Endo II transcript levels (quartiles) relative to 10 year distant metastasis-free survival for HER2+ lymph node positive patients (n=64). Median distant metastasis-free survival for high Endo II was 10.5 months, and 46 months for low Endo II cohort. **(B)** Kaplan-Meier plot for high or low Endo II transcript levels (quartiles) relative to 10 year relapse-free survival of chemotherapy-treated HER2+ patients (n=93). Median relapse-free survival was 10.5 months for high Endo II, and 46 months for low Endo II transcript levels. **(C)** Kaplan-Meier plot for high or low Endo II transcript levels (quartiles) relative to 10 year relapse-free survival of lymph node negative HER2+ patients (n=103). **(D)** Kaplan-Meier plot for high or low Endo II transcript levels (quartiles) relative to 10 year relapse-free survival of HER2+ patients, regardless of node status (n=252). **(E)** Kaplan-Meier plot for high or low Lamellipodin transcript levels (median) relative to 10 year relapse-free survival of lymph node positive HER2+ patients (n=100).

**
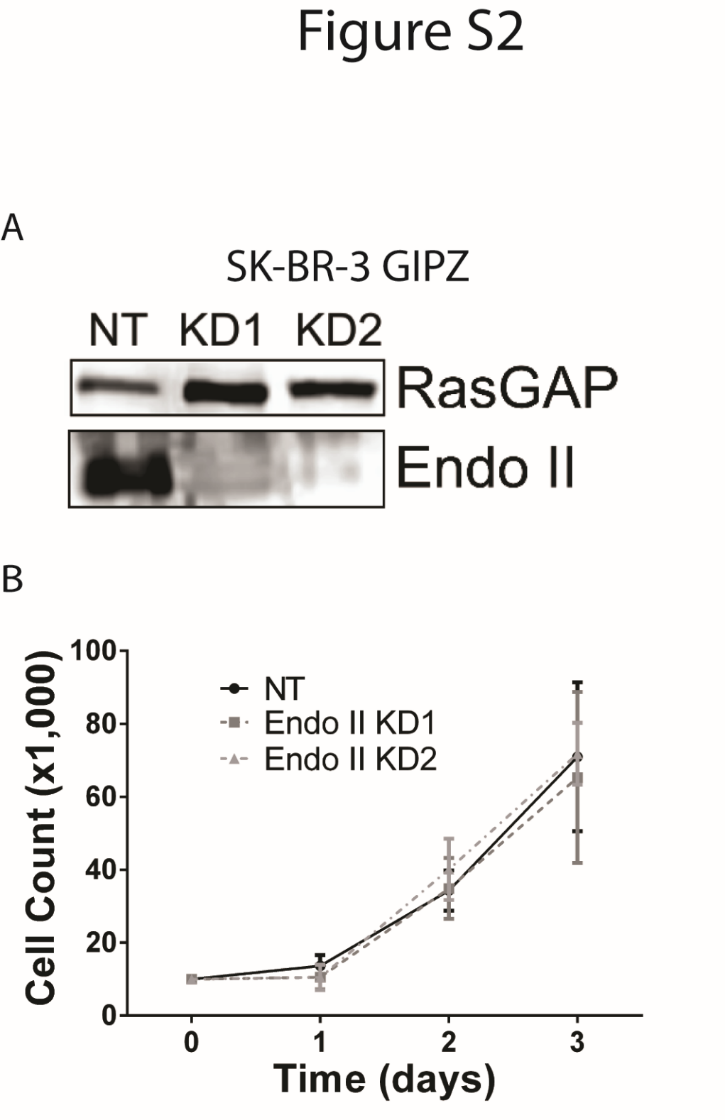
**

**Figure S2** **Endo II silencing in SK-BR-3 cells.** **(A)** GIPZ-based lentiviruses expressing non-targeting (NT) or Endo II-specific shRNAs were used to transduce SK-BR-3 cells and stable cell pools established. Lysates were subjected to immunoblot with Endo II and p120 RasGAP (as a loading control). **(B)** Graph depicts the growth rates of SK-BR-3 NT control and Endo II KD cells over a 3 day period.


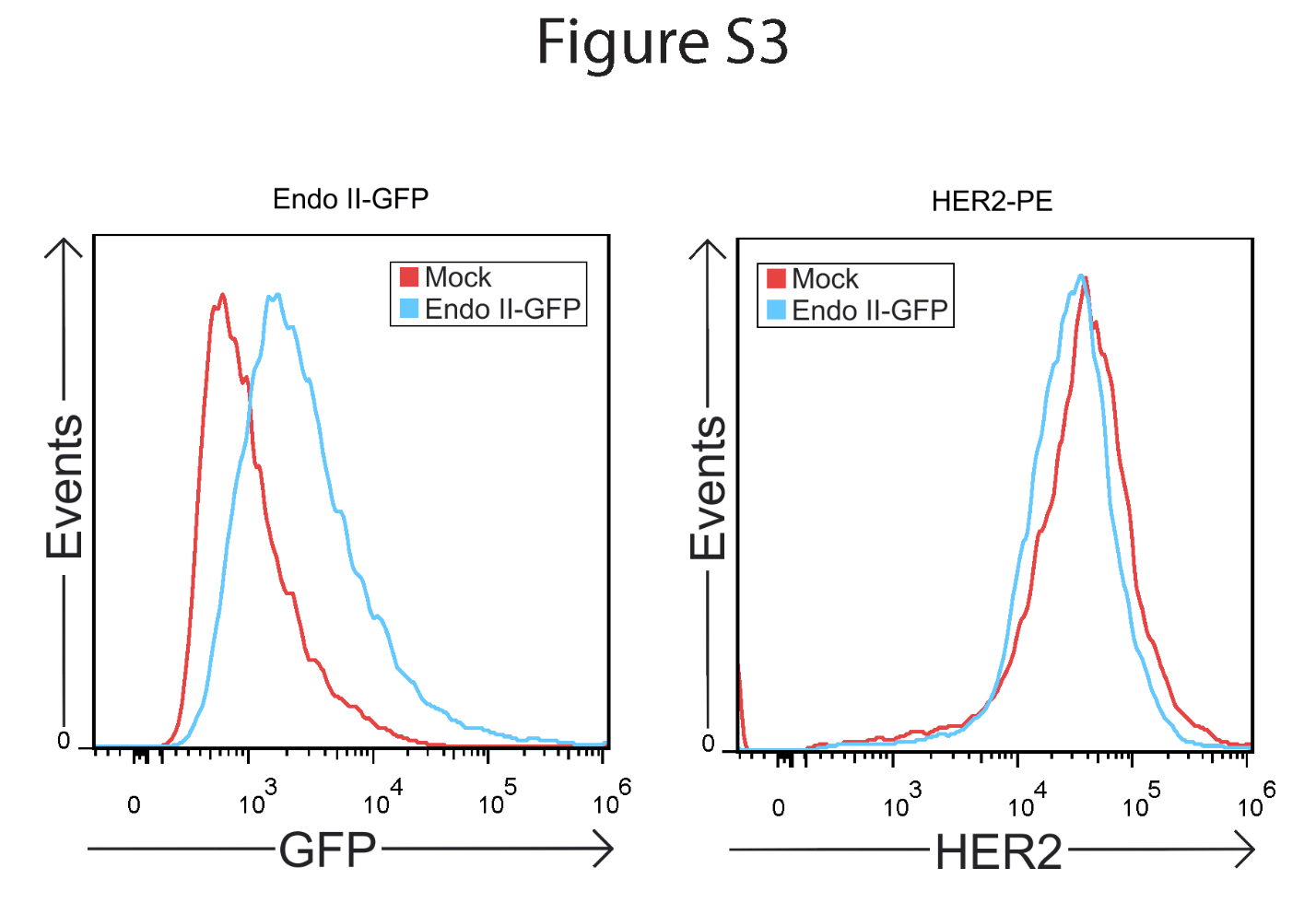


**Figure S3** **Endo II overexpression leads to reduced surface HER2.** BT-474 cells were transfected with pEGFP-Endo II (or mock transfected) and grown in serum conditions. After 48 hours, cells were incubated on ice, washed with cold PBS, followed by addition of PE-conjugated HER2 antibody, and analyzed by flow cytometry. Representative histograms are shown for GFP (left) and HER2 levels (right) in transfected BT474 cells.

**

Figure S4** **Endo II promotes EGF-induced Akt activation in SK-BR-3 cells**. **(A)** Serum-starved SK-BR-3 NT and Endo II KD cells were treated with or without EGF (100 ng/ml) for the indicated times (0-16 minutes). Lysates were subjected to immunoblot with phospho-specific antibodies to EGFR, Akt and Erk (along with pan reactive control antibodies). **(B)** Graphs depict the relative effect of EGF treatment on phosphorylation of the indicated proteins based on densitometry analysis from repeated experiments (significant differences between NT and Endo II KD indicate * *P*<0.05, ** *P*<0.01, *** *P*<0.001).


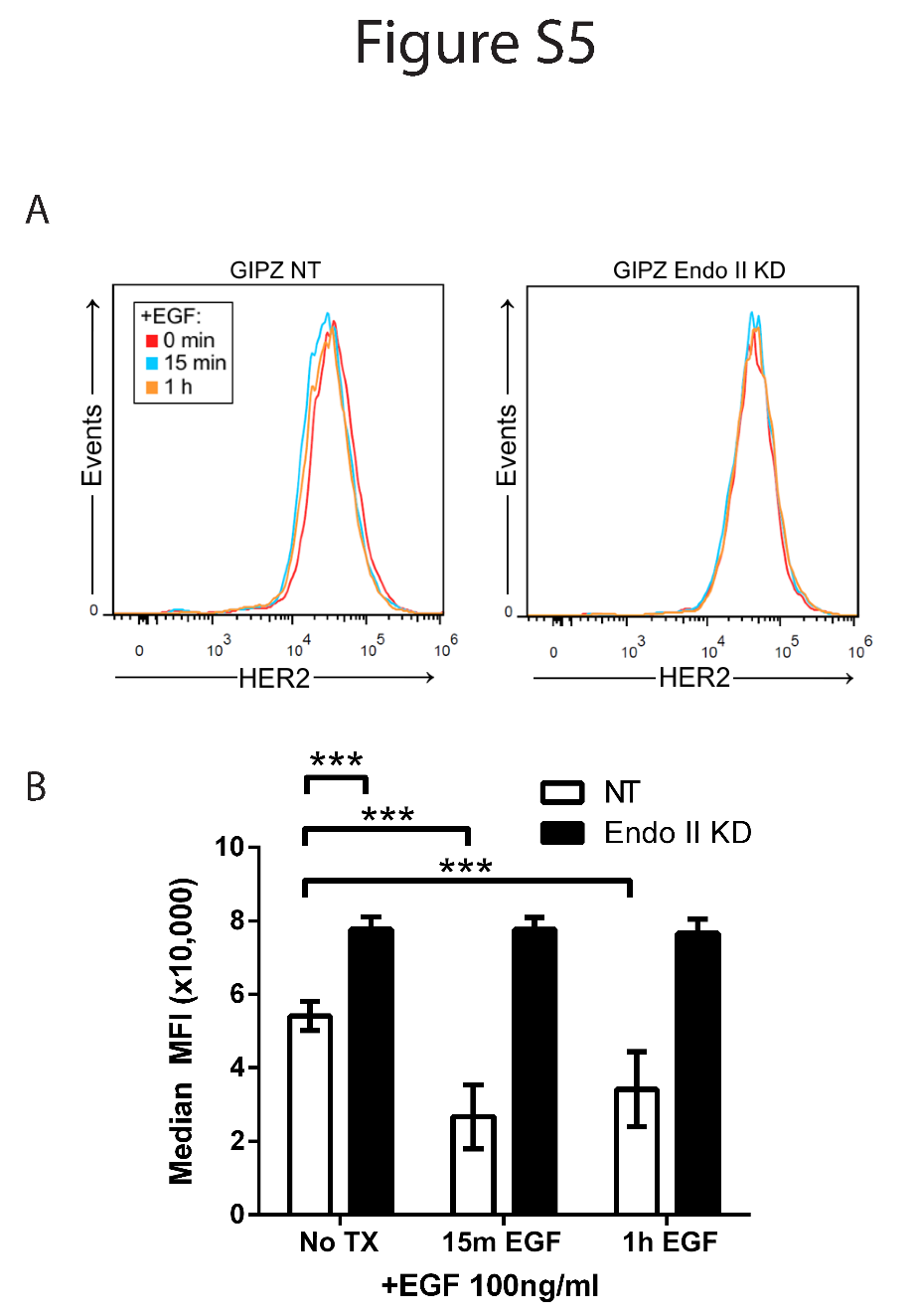


**Figure S5 Endo II promotes HER2 internalization in HCC1954 cells**. **(A)** Flow cytometry analysis was performed to measure surface HER2 levels in HCC1954 NT and Endo II KD cells treated with or without EGF for the indicated times and stained with PE-conjugated anti-HER2. Representative histograms are shown. **(B)** Graph depicts the shift in HER2 surface levels (MFI) in response to EGF treatment of SK-BR-3 NT and Endo II KD cells, and results are from repeated experiments.





**Figure S6 Endo II promotes SK-BR-3 cell motility.** **(A/B)** Transwell migration assays were performed with SK-BR-3 NT and Endo II KD1 and KD2 cells using transwell inserts and migration towards media supplemented with 10% serum (**A**) or 50ng/ml EGF (**B**). Graphs depict pooled results from repeated experiments (** *P*<0.01, *** *P*<0.001).





**Figure S7 Endo II silencing has no overt effects on HER2+ tumor growth.** Mammary orthotopic tumor xenograft assays were performed using Rag2^-/-^:IL2Rγc^-/-^ mice injected in the mammary fat pad with 1.5 x10^6^ HCC1954 NT or Endo II KD cells (n=8/group from 2 separate experiments). Tumors were harvested after 10 weeks and graph depicts the tumor mass for each group, with no significant differences observed.


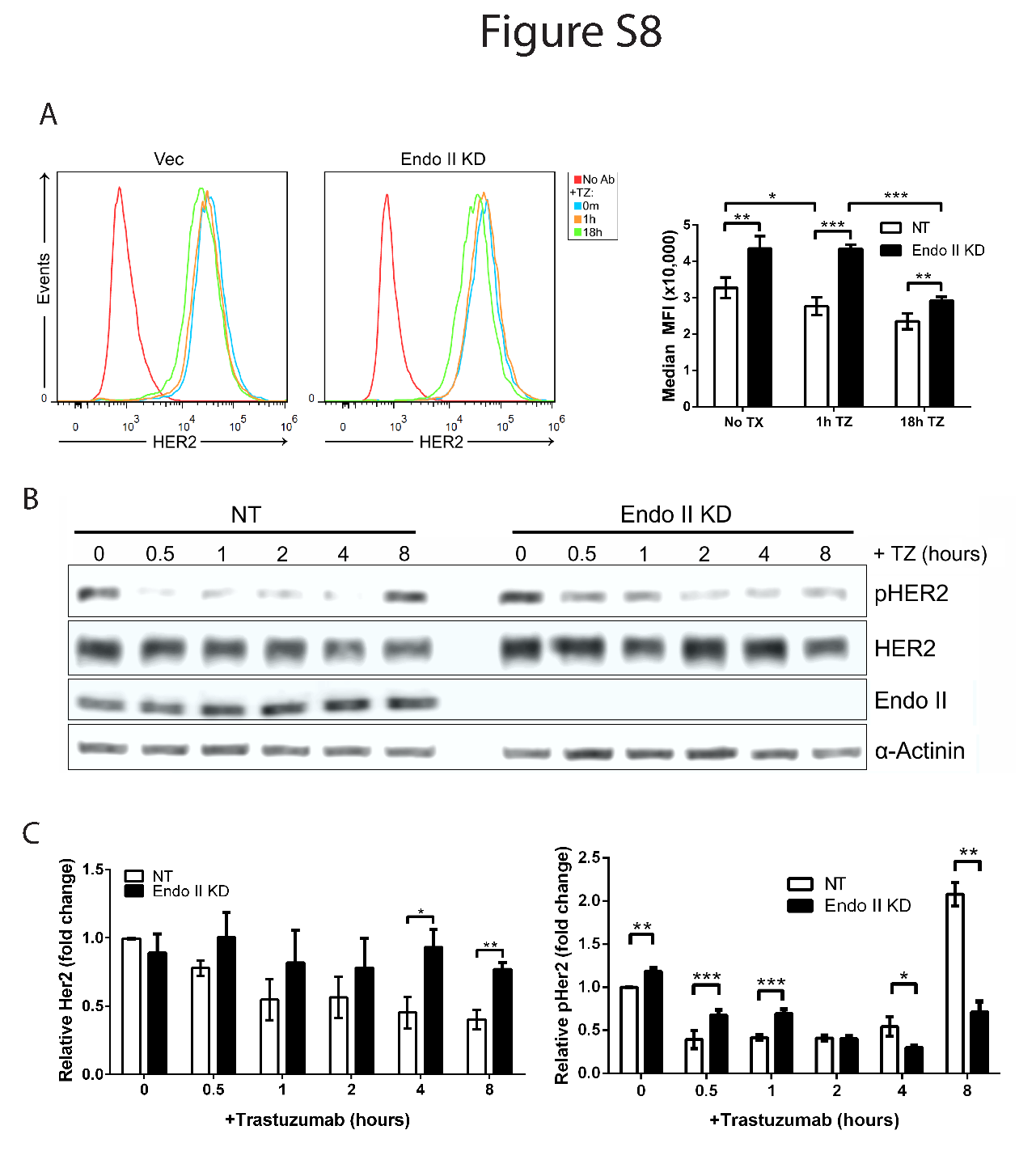
**Figure S8**  **Endo II promotes HER2 internalization and downregulation following Trastuzumab treatment of HCC1954 cells.** **(A)** Flow cytometry analysis of surface HER2 levels following Trastuzumab (TZ) treatment of HCC1954 Vec and Endo II KD cells for the indicated times. TZ treatment effects on surface HER2 levels (MFI) are shown for 3 independent experiments (* *P*<0.05, ** *P*<0.01, *** *P*<0.001). **(B)** Lysates from HCC1954 NT or Endo II KD cells treated with TZ (84 µg/ml) for the indicated times (0-8 hours) were subjected to immunoblot with antibodies to the indicated proteins (α-Actinin served as a loading control). **(C)** Densitometry was performed to quantify TZ treatment effects on HER2 levels (relative to α-actinin) and phospho-HER2 (pHER2, relative to pan HER2). Graphs represent results from 3 independent experiments (* *P*<0.05, ** *P*<0.01, *** *P*<0.001).


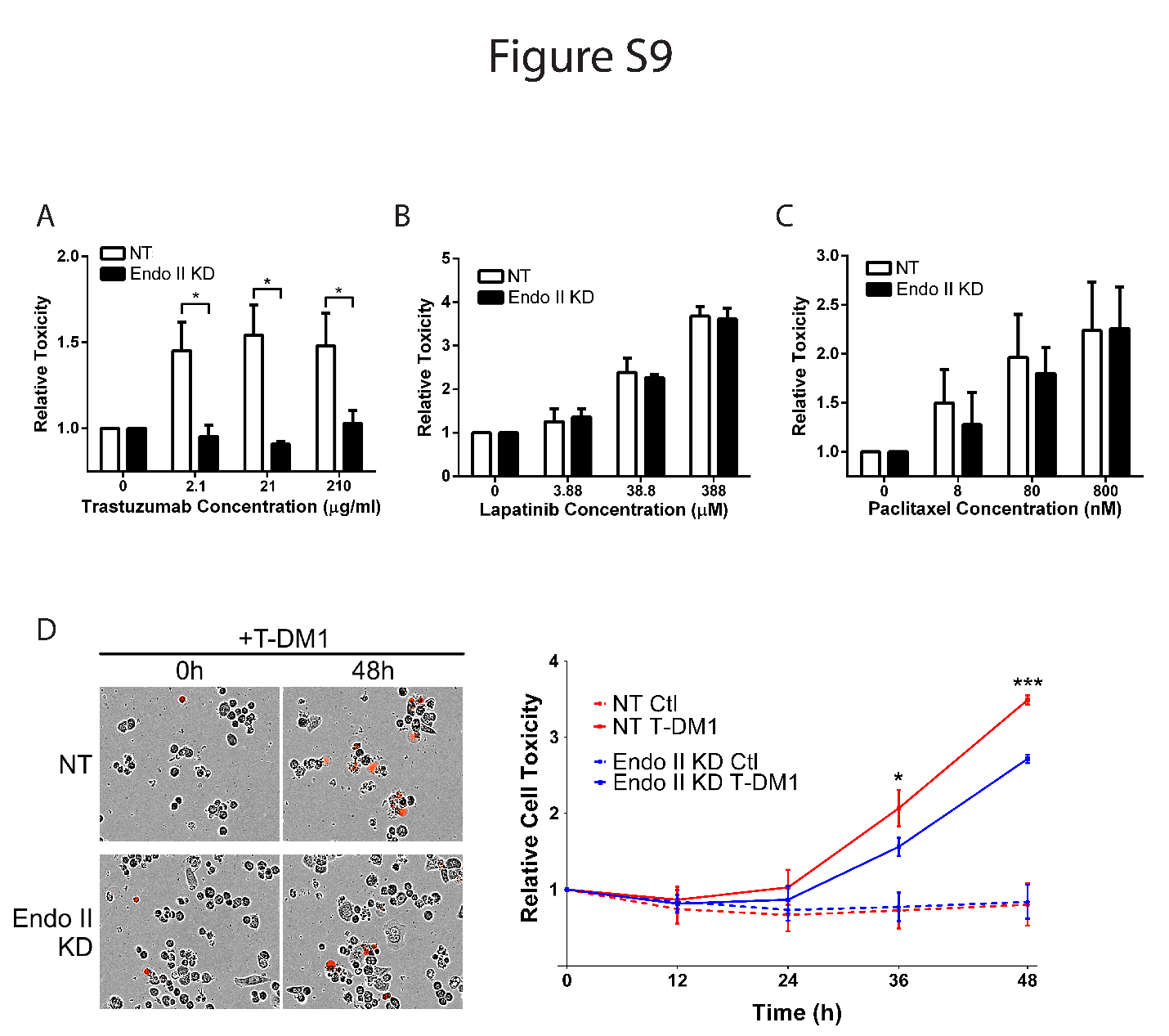


**Figure S9** **Endo II increases cytotoxicity upon treatment with Trastuzumab, but not with Lapatinib, in HER2+ cancer cells.** **(A-C)** Cytotox Glo cytotoxicity assays were performed on HCC1954 NT and Endo II KD cells treated with the indicated doses of Trastuzumab (**A**), Lapatinib (**B**), or Paclitaxel (**C**) after 48 hours. Relative toxicity values are based on luminescence in treatment sample compared to the untreated controls with results pooled from 3 separate experiments (* *P*<0.05). **(D)** Endo II increases T-DM1 induced cytotoxicity of SK-BR-3 cells. SK-BR-3 NT and Endo II KD cells seeded in a 96-well plate were treated with or without T-DM1 (50 ng/ml) in media supplemented with Propidium Iodide (PI) compared to control (Ctl; no drug added). Following real-time imaging, the relative increase in cytotoxicity (PI+ cells relative to GFP+ cells) was analyzed over 48 hours for 3 experiments performed in triplicate. Representative merged images of red channel (PI) and brightfield are shown on the left. The graph on the right indicates mean values (± SEM) from 3 experiments (significant differences between NT and Endo II KD cells treated with T-DM1 are denoted by * P<0.05; *** *P*<0.001).





**Figure S10** **Endo II overexpression results in increased sensitivity to T-DM1.** BT-474 were transiently transfected with pEGFP-Endo II (Endo-GFP), or mock transfected, and after 48 hours cells were seeded in 96 well plates for treatment with 84 µg/ml Trastuzumab (TZ), or 50 ng/ml T-DM1 or left untreated (Ctl). Cytotoxicity was measured in media containing PI using an IncuCyte ZOOM system over 108 hours. Graph depicts the relative cell toxicity measured by ratio of PI+ cells to total cells relative to time zero (significant differences between Mock and Endo-GFP cells treated with T-DM1 are denoted by *** *P*<0.001).
